# Profiling for Bioactive Peptides and Volatiles of Plant Growth Promoting Strains of the *Bacillus subtilis* Complex of Industrial Relevance

**DOI:** 10.3389/fmicb.2020.01432

**Published:** 2020-06-30

**Authors:** Pascal Mülner, Elisa Schwarz, Kristin Dietel, Helmut Junge, Stefanie Herfort, Max Weydmann, Peter Lasch, Tomislav Cernava, Gabriele Berg, Joachim Vater

**Affiliations:** ^1^ABiTEP GmbH, Berlin, Germany; ^2^Institute of Environmental Biotechnology, Graz University of Technology, Graz, Austria; ^3^ZBS6: Proteomics and Spectroscopy, Robert Koch-Institut, Berlin, Germany

**Keywords:** plant growth promoting rhizobacteria, bioactive peptides, volatiles, MALDI-TOF mass spectrometry, GC-MS, field trials

## Abstract

Plant growth promoting rhizobacteria attain increasing importance in agriculture as biofertilizers and biocontrol agents. These properties significantly depend on the formation of bioactive compounds produced by such organisms. In our work we investigated the biosynthetic potential of 13 industrially important strains of the *Bacillus subtilis* complex by mass spectrometric methodology. Typing of these organisms was performed with MALDI-TOF mass spectrometry followed by comprehensive profiling of their bioactive peptide products. Volatiles were determined by gas chromatography-mass spectrometry. Representative products of the members of the *B. subtilis* complex investigated in detail were: the surfactin familiy (surfactins, lichenysins, pumilacidins); the iturin family (iturins, mycosubtilins and bacillomycins); plantazolicin and the dual lantibiotics lichenicidins, as well as a wide spectrum of volatiles, such as hydrocarbons (alkanes/alkenes), alcohols, ketones, sulfur-containing compounds and pyrazines. The subcomplexes of the *B. subtilis* organizational unit; (a) *B. subtilis/Bacillus atrophaeus*; (b) *B. amyloliquefaciens/B. velezensis*; (c) *B. licheniformis*, and (d) *B. pumilus* are equipped with specific sets of these compounds which are the basis for the evaluation of their biotechnological and agricultural usage. The 13 test strains were evaluated in field trials for growth promotion of potato and maize plants. All of the implemented strains showed efficient growth stimulation of these plants. The highest effects were obtained with *B. velezensis, B. subtilis*, and *B. atrophaeus* strains.

## Introduction

Plant growth promoting rhizobacteria (PGPR) and endophytes attain increasing importance as efficient biofertilizers, biostimulants and biocontrol organisms for a sustainable and enviromentally friendly alternative to conventional agriculture (Saharan and Nehra, 2011; Hardoim et al., 2015). PGPR establish an mutualistic symbiosis by interacting with plant roots in the rhizosphere or colonizing plant vessels and organs as endophytes and are enhancing plant growth through production of secondary metabolites, phytohormones and facilitation of nutrient uptake (Barea et al., 2005; Drogue et al., 2012).

In contrast to agrochemicals, these biological compounds are biodegradable and only produced at site (Lugtenberg and Kamilova, 2009). Among other plant associated microorga-nisms, many PGPR are capable of triggering plant defense mechanisms (van Loon and Glick, 2004). This elicitation of innate immunity responses of plants is called induced systemic resistance (ISR) (van Loon et al., 1998). ISR is induced by dynamic chemical communication between plants and bacteria (van Loon, 2007; Farag et al., 2013). Numerous PGPR produce a variety of antimicrobial compounds to inhibit phytopathogens (Ongena and Jacques, 2008; Pérez-García et al., 2011). Such compounds are frequently produced non-ribosomally by specific multienzyme systems. In addition, also a wide spectrum of bioactive peptides of ribosomal origin, such as lantibiotics and bacteriocines are recruited for plant protection. Prominent microorganisms with such abilities include members of the *B. subtilis* complex, *Paenibacillus* as well as *Pseudomonas* strains (Lim et al., 1991; Hong and Meng, 2003; Peighami-Ashnaei et al., 2009; Asari et al., 2016; Rybakova et al., 2016).

Two specific effects of such organisms are in focus for future agricultural developments, (a) their ability to stimulate plant growth and performance, and (b) their biocontrol effects to protect their plant hosts against deleterious effects of phytopathogenic competitors as well as against abiotic stress (Berg, 2015). PGPR organisms are qualified to increase plant harvest significantly and to protect plants against harmful bacteria, fungi, nematodes and insects. For both effects their biosynthetic potential to produce a broad spectrum of secondary metabolites, such as bioactive peptides, polyketides and hybrids thereof as well as small volatile organic compounds (VOCs) (Audrain et al., 2015; Kanchiswamy et al., 2015; Panpatte et al., 2017) are of outmost importance. VOCs produced by PGPR exhibit a high chemical diversity and play a significant role in the stimulation of plant growth, control of plant pathogens, regulation of phytohormone synthesis and metabolism as well as induction of systemic resistance against plant diseases (Kloepper et al., 2004; Werner et al., 2016; Bailly and Weisskopf, 2017; Wu et al., 2018). Therefore, they are of high value for plant productivity and health.

The plant-growth enhancing activity of members of the *B. subtilis* complex are of great industrial relevance. The four industrially important species *Bacillus subtilis*, *Bacillus amyloliquefaciens, Bacillus licheniformis*, and *Bacillus pumilus* are part of the *Bacillus subtilis* complex (Fritze, 2004; Fan et al., 2017). They represent a family of genetically closely related organisms. Since a few decades the exploration of the biosynthetic potential of members of the *B. subtilis* complex for the production of secondary metabolites is under current investigation (Kowall et al., 1998; Bonmatin et al., 2003; Vater et al., 2003; Koumoutsi et al., 2004; Argeulles-Arias et al., 2009; Monaci et al., 2016; Zhao and Kuipers, 2016; Tahir et al., 2017a). Studies on the nature and application of lipopeptides and volatiles formed by such rhizobacteria are of particular interest for biocontrol as well as productivity and health of plants. This research field is presently under intensive reviewing (Caulier et al., 2019; Dunlap et al., 2019; Olishevska et al., 2019; Rabbee et al., 2019; Keswani et al., 2020) with a focus on the biosynthetic equipment of the members of the *B. subtilis* complex for bioactive compounds.

Both phenotypic and phylogenetic approaches, such as 16S rRNA sequencing, led to difficulties in distinguishing different species of the *Bacillus subtilis* group (Rooney et al., 2009). Recently, it was demonstrated by phylogenomic analysis that the *B. subtilis* complex can be organized into four subcomplexes (Fan et al., 2017; Dunlap et al., 2019). Subcomplex I (*subtilis*) diverges into two main branches comprising the closely related *B. subtilis* and *B. atrophaeus* strains. Subcomplex II (*amyloliquefaciens*) is divided into *B. amyloliquefaciens*, *subspecies amyloliquefaciens* and *B. amyloliqufaciens*, *subspecies plantarum* which recently has been renamed as *B. velezensis*. Subcomplexes III and IV are represented by *B. licheniformis* and *B. pumilus* strains, respectively.

Our research was focussed on the investigation of the biosynthetic capacity of 13 industrially important PGPR-strains of the *B. subtilis* complex using modern mass spectrometric techniques of high resolution and sensitivity. In particular, MALDI-TOF MS and GC-MS are qualified for this task as the methods of choice, both for rapid typing and characterization of the investigated organisms as well as for efficient exploration of their biosynthetic potential. Here we present a comprehensive mass spectrometric study of the bioactive peptides and volatile compounds produced by these strains which play an important role in their plant growth promoting abilities. The increase of the produce in the harvest of potatoes and maize induced by them was studied in field trials.

## Materials and Methods

### Materials

The matrices α-cyanohydroxycinnamic acid (CCA) and dihydroxy-benzoic acid (DHB) used for MALDI-TOF MS were obtained from Bruker (Bremen, Germany). Acetonitrile (ACN, HPLC grade) was purchased from Merck (Darmstadt, Germany), trifluoroacetic acid (TFA) from Sigma-Aldrich (Deisenhofen, Germany). Type strains of the *B. subtilis* complex were available from DSM (Deutsche Sammlung von Mikroorganismen, Braunschweig, Germany).

### Cultivation of Organisms

For the preparation of surface extracts the investigated organisms of the *B. subtilis* complex were grown on Agar plates using three cultivation media: (a) Lysogeny Broth (LB) medium; (b) Landy medium (Landy et al., 1948) and (c) TSA-medium [composition per L: 15 g casein peptone (pancreatic), 5 g soya peptone, 5 g sodium chloride, 15 g agar per L deionized water, final pH = 7.3] solidified with 1.5% agar in petri dishes for 24; 48, and 72 h at 30°C. In addition, liquid fermentations were carried out in 100 ml Erlenmeyer flasks at 30°C and 200 rpm in an orbital shaker (Buchler, Germany) to detect products released by the strains into the culture medium. Cells were harvested after 8; 10; 12; 24; 48; and 72 h of incubation. The PGPR were cultivated both in Landy and TSA medium.

### Sample Preparation

In order to obtain complete profiles of bioactive peptides produced by the investigated PGPR of the *B. subtilis* complex, their products were detected by MALDI-TOF MS (a) in surface extracts of cells picked either from agar plates or harvested from liquid cultures by centrifugation for 10–20 min at 15000 rpm, (b) in culture supernatants after growth for 8; 10; 12; 24; 48; and 72 h and (c) after disintegration by solubilization with 80% acetonitrile.

For (a) a wire loop of cell material was picked, suspended in 50 μl 50% acetonitrile/0.1% TFA and extracted for 15 min by vigorous vortexing. Finally, cells were spun down at 15000 rpm for 10 min.

### Mass Spectrometric Identification of the Investigated PGPR Organisms

PGPR strains were identified by MALDI-TOF MS according to the procedure developed by Lasch et al. (2008). A wire loop of cell material was picked from Agar plates and transferred into 20 μl of water and mixed with 80 μl of pure TFA (Uvasol grade; Merck, Darmstadt, Germany). The suspension of bacteria was incubated under gentle mixing for 30 min at room temperature until cells were completely solubilized. Then 10 μl of the suspension were diluted 1:10 with bidistilled water adjusting a final TFA-concentration of 8%. Now the samples get turbid, because a part of the solubilized material becomes insoluble and precipitates. Two μl of the turbid suspension were mixed with 2 μl of a saturated solution of the CCA matrix for MALDI-TOF mass spectrometric analysis. Mass spectra were generated in linear mode in the mass range of 1–10 kDa. The effects of 3 × 3000 laser shots were accumulated. Data acquisition and analysis were carried out using the MALDI-Biotyper software package provided by Bruker (Bremen, Germany) and a Matlab-based software (Microbe MS). For MALDI-TOF MS identification analysis, samples were processed, as outlined in Lasch et al. (2008, 2015). For evaluation the obtained mass spectra were smoothed and baseline-corrected.

### Profiling of Bioactive Peptides by MALDI-TOF MS

Bioactive peptides of the tested PGPR of the *B. subtilis* complex were detected and identified by MALDI-TOF MS, as outlined previously (Vater et al., 2017, 2018). A Bruker Autoflex Speed TOF/TOF mass spectrometer (Bruker Daltonik, Bremen, Germany) was used with smartbeam laser technology using a 1 kHz frequency-tripled Nd-YAG laser (λ_ex_ = 355 nm). Samples (2 μl) of surface extracts and culture supernatants were mixed with 2 μl matrix solution (a saturated solution of α-hydroxy-cinnamic acid in 50% aqueous ACN containing 0.1% TFA), spotted on the target, air dried and measured. Mass spectra were obtained by positive-ion detection in reflector mode. Monoisotopic masses were obtained. Parent ions were detected with a resolution of 10,000.

Sequence analysis of the lipopeptide products was performed by MALDI-LIFT-TOF/TOF mass spectrometry in laser induced decay (LID) mode (Suckau et al., 2003). The product ions in the LIFT-TOF/TOF fragment spectra were obtained with a resolution of 1000.

The mass spectrometry results did not allow optical isomers to be distinguished. Therefore, the configurations of the amino acid components were not indicated.

### Profiling of Volatiles by GC-MS

The PGPR strains were grown in 20 ml headspace vials (75.5 × 22.5 mm; Chromtech, Idstein, Germany) filled with 8 ml of nutrient agar (Sifin Diagnostic GmbH, Berlin, Germany) and mVOCs measured according to the protocol of Cernava et al. (2015). Equal distribution was assured by parallel application of cell material in three vials per organism. After 24 h of incubation at 30°C, the volatiles accumulating inside the vials were measured by headspace-solid phase microextraction (HS-SPME) GC-MS. Compound separation and detection was performed on a system combining a gas chromatograph 7890A with a quadrupol mass spectrometer 5975C (Agilent Technologies, Waldbronn, Germany). A solid phase microelution (SPME) fiber consisting of divinylbenzene/carboxen/polydimethylsiloxane (DVB/CAR/PDMS) was used for sampling (Sigma-Aldrich, St. Louis, United States). Head space (HS) samples entered a (5%-phenyl)methylpolysiloxane column (60 m × 0.25 mm) i.d.; 0.25 μm film thickness (DB-5MS; Agilent Technologies, Waldbronn, Germany). Subsequently, electron ionization with 70 eV and detection in the mass range 25–350 Da were performed. The fiber was desorbed at 200°C for 8 min to eliminate potential residues before initial measurements. 270°C was chosen for the inlet temperature. The temperature gradient of the column was maintained at 70°C for 1.5 min, raised to 200°C at a rate of 16°C/min and finally kept at 200°C for 0.5 min. The helium flow rate was adjusted to 1.2 ml/min. For identification of the volatiles the received spectra were matched to the NIST (National Institute of Standards and Technology) Mass Spectral Database 14 via MS Search 2.2. Additionally, the Kovats Indices of all compounds were calculated and verified with corresponding entries in the online database Chemspider.^1^

### Field Trial Experiments

The field trials were carried out in field at D-18190 Gross Lüsewitz, Mecklenburg-Vorpommern (Germany) (54°04′44.7′′N 12°21′45.0′′E) with slightly loamy sand (total N 21, P 9.6, K 1.0 and Mg 4.0 mg/100 g soil, pH 5.2), the typical soil type of the area. The experimental field was divided into plots with four replicates per treatment in a randomized block design. For potato the plots were 21 square meter (7 m × 3 m) and for maize 18 square meter (3 m × 6 m). The distance between the rows were 75 cm with 4 rows per plot. Tubers of *Solanum tuberosum* cv. Goldmarie (Norica Nordring-Kartoffelzucht- und Vermehrungs-GmbH) were treated with the microbial inoculum (0.5 l/ha or 1.0 l/ha in 300 l water via in-furrow spray at planting using a Parzellenspritze PL1, AirMix 110-03, 3.2 kp/cm^2^). Seeds of *Zea mays* cv. Colisee (KWS Saat SE & Co. KG) were treated with the microbial inoculum (0.5 l/ha or 1.0 l/ha) in 300 l water via in-furrow spray at sowing (Parzellenspritze PLL1, type of nozzle: AirMix 110-03; operating pressure 3.2 kp/cm2). The microbial inoculum of each strain contained 2.5E+10 cfu/ml. Mineral fertilizer was added based on a chemical analsis of the soil and was applied before planting (N 121.5 kg/ha, P 37.5 kg/ha, K 127.5 kg/ha). The potatos were planted on 16th of May and harvested on 20th of September (BBCH99). The maize was sown on 4th of June and harvested on 18th of October (BBCH86). For yield determination only they were harvested and weight was measured. The effect of microbial inoculum on potato or maize growth was calculated based on assessed weight of the individual tubers of all plants of the two middle rows of each plot. The mean of the four replicates was calculated and the standard deviation was determined.

## Results

### Identification and Characterization of the Investigated PGPR Strains by MALDI-TOF MS

In our work we investigated 13 bacterial test strains of industrial relevance. In the first step these organisms were classified by MALDI-TOF mass spectrometry. MALDI-TOF disintegration mass spectra were generated and evaluated using the Bruker Daltonik MALDI Biotyper (Bruker Daltonik, Bremen, Germany) and a Matlab-based software (Microbe MS) comprising mass spectra of a wide spectrum of bacterial strains including highly pathogenic organisms (Lasch et al., 2015). In this way, typing of the investigated PGPR strains was achieved. As demonstrated in Table 1 all of them were distributed among the four subcomplexes of the *B. subtilis* species complex. The MALDI-TOF mass spectra of these strains were correlated with those of the corresponding type strains.

**TABLE 1:** Profiles of bioactive peptides of the investigated PGPR -strains.

**Strain**	**Classification by MALDI-TOF MS**	**Bioactive peptides**
**Subcomplex a: *B. subtilis* and *B. atrophaeus***		
168^T^	*B. subtilis*	–
DSM 32873	*B. subtilis*	Surfactin, fengycin, plantazolicin
Sc-S7	*B. subtilis*	Surfactin, fengycin
DSM 7264^T^	*B. atrophaeus*	Surfactin, mycosubtilin, fengycin *m/z* = 2754.6 Da
DSM 32285	*B. atrophaeus*	Surfactin, iturin C, fengycin
DSM 29418	*B. atrophaeus*	Surfactin, mycosubtilin, fengycin, *m/z* = 2755.2 Da
**Subcomplex b: *B. amyloliquefaciens* and *B. velezensis***		
DSM 7^T^	*B. amyloliquefaciens*	Surfactin, iturin A
DSM 32875	*B. amyloliquefaciens*	Surfactin, iturin A
DSM 23117^T^ (FZB 42)	*B. velezensis*	Surfactin, bacillomycin D, fengycin, plantazolicin
DSM 29399 (FZB 45)	*B. velezensis*	Surfactin, bacillomycin L, fengycin
Rs-MS87	*B. velezensis*	Surfactin, bacillomycin D, fengycin, plantazolicin
Sc-K143	*B. velezensis*	Surfactin, bacillomycin D, fengycin, plantazolicin
**Subcomplex c: *B. licheniformis***		
DSM 13^T^	*B. licheniformis*	Lichenicidin, *m/z* = 1080.6 Da
DSM 32874	*B. licheniformis*	Lichenysin, m/z = 1796.4 Da
M18	*B. licheniformis*	Lichenysin, m/z = 1796.4 Da
**Subcomplex d: *B. pumilus***		
DSM 27^T^	*B. pumilus*	Plantazolicin
Abi 11	*B. pumilus*	Pumilacidin, plantazolicin
Rs-Ts276	*B. pumilus*	Pumilacidin, plantazolicin, *m/z* = 1851.2/1982.3

**FIGURE 1:**
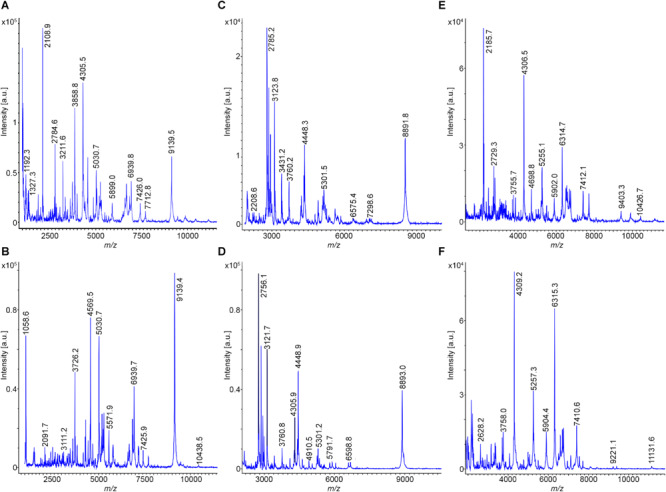
MALDI-TOF mass spectra for typing of *B. subtilis* 168^T^ **(A)** and DSM 32873 **(B)**; *B. atrophaeus* DSM 32285 **(C)** and DSM 29418 **(D)** as well as *B. amyloliquefaciens* DSM 7^T^ **(E)** and DSM 32875 **(F)**. Cell material was picked from agar plates, solubilized with 80% trifluoroacetic acid and processed as described under Methods. Mass spectra were taken in the mass range from 1–10 kDa.

*Bacillus subtilis* 168^T^ is the type strain for *B. subtilis*; DSM 7264^T^ for *B. atrophaeus*; DSM 7^T^ for *B. amyloliquefaciens*, subsp. *amyloliquefaciens*; DSM 23117^T^ (FZB42) for *B. amyloliquefaciens*, subsp. *plantarum*, now designated as *B. velezensis*; DSM 13^T^ for *B. licheniformis* and DSM 27^T^for *B. pumilus*. Prominent mass peaks showing high intensities were taken as biomarkers for strain identification from the MALDI-TOF disintegration mass spectra of the different types of organisms (Figure 1 and Supplementary Figures S1, S2). They were related to the corresponding peaks in the mass spectra of the reference strains. In each subcomplex at least six of such biomarkers were selected for rapid typing of members of the *B. subtilis* complex. The obtained biomarkers are summarized in Table 2.

**TABLE 2:** Biomarker for the identification of members of the *B. subtilis* complex.

**Type of organism**	**Biomarker selected by MALDI-TOF MS (*m/z*)**
*B. subtilis*	4305.4/9138.7/5030.5/4568.9/3858.5/3725.5
*B. atrophaeus*	2771.3/2867.0/3122.8/3760.5/4448.6/8892.4
*B. amyloliquefaciens*	6315.0/4307.9/2192.3/5256.2/5903.2/7411.4
*B. velezensis*	6152.8/3076.3/4306.0/4988.9/7526.4/9407.3
*B. licheniformis*	5895.4/2948.6/4306.1/3252.6/5253.0/7086.6
*B. pumilus*	6022.7/6054.1/6092.6/7231.0/4306.8/6885.2/3616.5/9376.9/9829.5

By evaluation of the MALDI-TOF mass spectra taken after complete disintegration of the bacterial cells by the Bruker and RKI data bases the following assignments of the investigated strains were obtained (see Table 1): DSM 32873 and Sc-S7 were classified as *B. subtilis* strains. DSM 32285 and 29418 were identified as *B. atrophaeus*. DSM 32875; DSM 23117^T^ (FZB42); DSM 29399 (FZB45); Rs-MS87 and Sc-K143 are members of the subcomplex *amyloliquefaciens*. Here a clear differentiation between subspecies *amyloliquefaciens* and subspecies *plantarum* (*B. velezensis*) was accomplished. The mass spectrum of DSM 32875 is different from the other strains and corresponds well with that of DSM 7^T^, the type strain of *B. amyloliquefaciens*, subsp. *amyloliquefaciens*, while the mass spectra of DSM 29399 (FZB45); Rs-MS87 and Sc-K143 correlate nicely with that of DSM 23117^T^ (FZB42) which is the type strain for *B. velezensis*. DSM 32874 and M18 fit into the subcomplex *B. licheniformis*. Their mass spectra correspond well with the *B. licheniformis* type strain DSM13^T^. Abi11 and Rs-Ts276 were identified as *B. pumilus* strains and related to the *B. pumilus* type strain DSM 27^T^. Our mass spectrometric classification of the investigated PGPR strains agrees with previous biochemical and genetic data.

### Profiling of the Investigated PGPR-Strains for Bioactive Peptides

After having classified our test strains as members of the *B. subtilis* complex the main aim of our work was focused on the exploration of their biosynthetic potential for the production of bioactive peptides and volatiles which play an important role in plant growth promoting and biocontrol activities of PGPR strains. The obtained profiles were related to those of commonly used type strains available from DSM. For this purpose product formation of both test and type strains was studied in a space- and time fashioned manner.

We initiated a comprehensive study of the bioactive peptides produced by our strains attributed to the *B. subtilis* complex in the mass range from 400 to 3500 Da.

In order to determine the complete production of bioactive compounds, three series of mass spectrometric tests were performed: MALDI-TOF mass spectra were taken (a) from surface extracts and (b) after complete disintegration of cells picked from agar plates as well as (c) from culture supernatants to detect products that were released into the culture medium. For tests a and b cells were grown on agar plates at 30°C for 24; 48; and 72 h using three cultivation media (Landy, LB and TSA). Surface extracts were obtained by extraction of cell material picked from the plates with 50% acetonitrile/0.1% TFA. For test b to detect products within cells and attached to their surface, cell material was disintegrated with 80% TFA and processed as indicated above. In addition, for test c the isolates were cultivated for 8–72 h either in the Landy- or TSA- medium at 30°C. Released products were detected by MALDI-TOF MS. Thus, product formation of the investigated PGPR strains of the *B. subtilis* complex was obtained in a space- and time fashioned manner. The obtained results were summarized in Table 1. All investigated strains produced the siderophore bacillibactin with mass numbers of [M + H,Na,K]^+^ = 883.4/905.4/921.4 which was preferentially detected in culture supernatants.

Structure elucidation of bioactive peptides was performed by fragment analysis using LIFT-MALDI-TOF/TOF MS (Suckau et al., 2003). Polyketide formation will be the subject of a forthcoming publication.

### Subcomplex a: *B. subtilis* and *B. atrophaeus*

**FIGURE 2:**
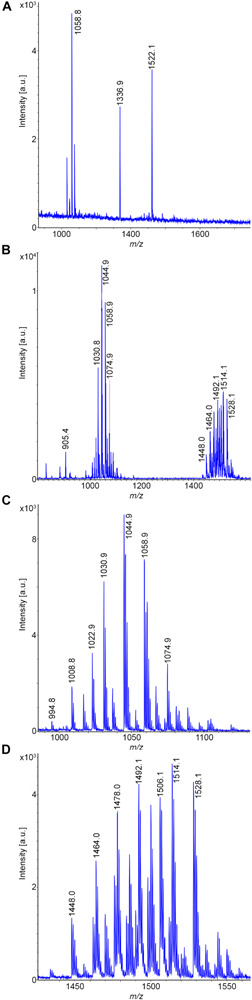
Detection of bioactive peptides produced by *B. subtilis* DSM 32873: **(A)** MALDI-TOF mass spectrum of a surface extract of this organism grown on TSA-agar. **(B)** MALDI-TOF mass spectrum of the culture supernatant of DSM 32873 grown in the Landy medium for 24 h at 30°C. **(C,D)** MALDI-TOF mass spectra of the surfactin- **(C)** and fengycin **(D)**-complex. C13–C15-surfactins and C14–C17-fengycins A and B were detected.

**FIGURE 3:**
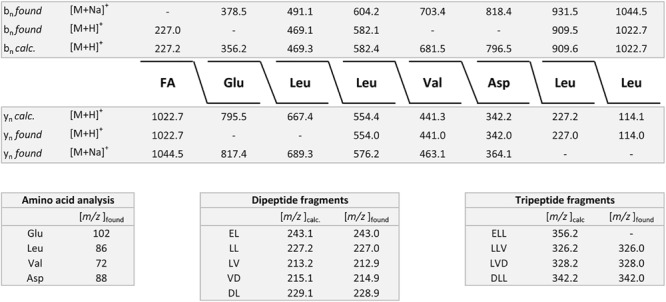
Mass spectrometric sequence determination of a C14-surfactin produced by *B. subtilis* DSM 32873 with parent ions [M + H,Na]^+^ = 1022.7 and 1044.5 Da derived from product ion patterns obtained by LIFT-MALDI-TOF/TOF fragment spectra. FA: CH_3_-(CH_2_)_10_-CHOH-CH_2_-CO-.

Two *B. subtilis* strains were investigated, DSM 32873 and Sc-S7. Bioactive peptides produced by strain DSM 32873 are demonstrated in Figures 2A–D. Figure 2A shows the MALDI-TOF mass spectrum of a surface extract of this organism using TSA as cultivation medium. It produces the surfactin and fengycin lipopeptide families which are formed non-ribosomally at multimodular enzyme complexes (Menkhaus et al., 1993; Steller et al., 1998). Prominent species were found for surfactin at *m/z* = 1058.8 and m/z = 1522.1 for fengycin, resp. The mass peak at *m/z* = 1336.9 can be attributed to plantazolicin, a highly condensed thiazole/oxazole modified microcin (TOMM) of ribosomal origin (Kalyon et al., 2011; Scholz et al., 2011). Plantazolicin was not found in the culture supernatant (Figure 2B). Surfactins and fengycins together with the siderophore bacillibactin were released into the culture medium, as is apparent from the mass spectrum of the culture supernatant, when DSM 32873 was grown for 24 h at 30°C in the Landy medium (Figure 2B). The mass spectra in Figures 2C,D display the surfactin and fengycin complexes in their full complexity. The observed mass peaks for surfactin can be assigned to the protonated forms and the alkali adducts of C13-C15 surfactins, resp. Fengycins represent the protonated species and alkali adducts of C14-C17 fengycins A and B, which differ in the amino acid component at position 6. Ala in the case of fengycin A is replaced by Val for fengycin B. Similar results were obtained for *B. subtilis* strain Sc-S7, but in contrast to DSM 32873 it did not produce plantazolicin. A specific feature of *B. subtilis* strains is the complete absence of iturin compounds. The product spectrum determined mass spectrometrically is in accordance with the product pattern derived from genome sequencing of *B. subtilis* strains (Fan et al., 2017; Harwood et al., 2018). Surfactin was identified mass spectrometrically by LIFT-MALDI-TOF/TOF fragment analysis. In Figure 3 the fragment pattern of C14-surfactin is demonstrated. The parent ions were [M + H,Na]^+^ = 1022.7 and 1044.5, respectively. Applying MALDI-TOF MS the alkali adducts of surfactin dominate in the mass spectra. The same feature was found for the fragment ions, particularly for the larger ones. Therefore, for sequence determination of surfactins both the protonated and sodiated product ions were available. From the product ions derived from the LIFT-MALDI-TOF/TOF fragment spectrum, the surfactin sequence was approached in several steps. In the low mass range *m/z* < 160 Da the immonium ions allow detection of the amino acid components of surfactin. From the observed di- and tripeptide fragments the complete lipopeptide sequence can be modeled. Finally, the protonated b_n_ and y_n_ – ions as well as their sodium adducts were collected and compared with the calculated values, from which in the upper part of Figure 3 the complete sequence of C14-surfactin was derived. In general, it was not possible to discriminate between Leu and Ile in our MALDI-LIFT-TOF/TOF studies for sequence elucidation of surfactin and other investigated bioactive peptides. Here, the assignment was performed according to literature data.

**FIGURE 4:**
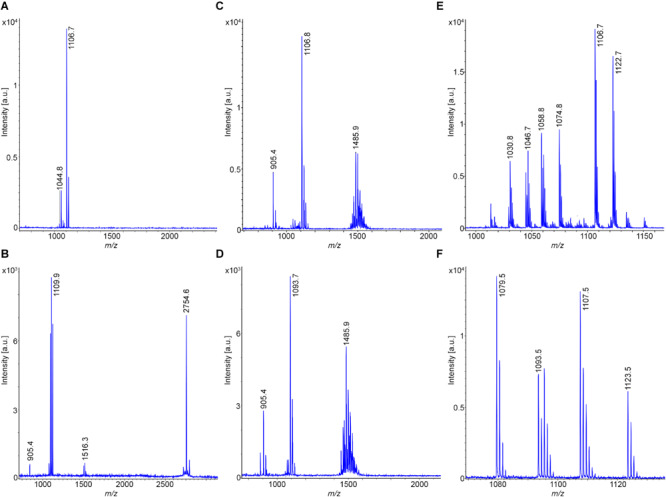
Detection of bioactive peptides produced by *B. atrophaeus* DSM 32285 and DSM 29418: **(A,B)** MALDI-TOF mass spectra of surface extracts of these strains. **(C,D)** MALDI-TOF mass spectra of culture supernatants of these strains grown for 24 h at 30°C in the Landy medium. **(E)** MALDI-TOF mass spectrum of the surfactin and iturin C complexes produced by strain DSM 32285. C13–C15 surfactins and C16–C19 iturins C were detected. **(F)** MALDI-TOF mass spectrum of the mycosubtilin complex produced by strain DSM 29418. C15-C17 mycosubtilins were detected.

**FIGURE 5:**
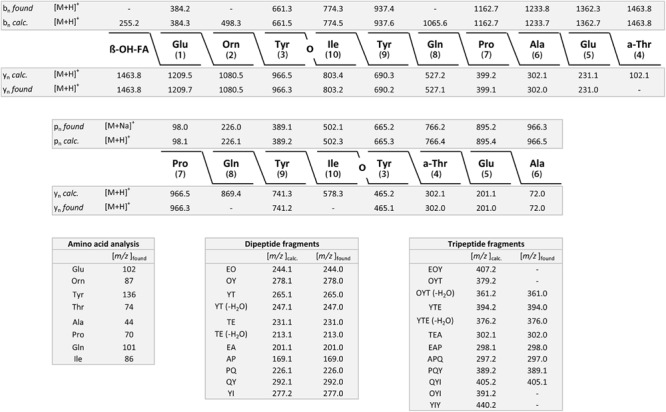
Mass spectrometric sequence determination of a C16-fengycin produced by DSM 7264^T^ with a parent ion [M + H]^+^ = 1463.8 derived from product ion patterns obtained by LIFT-MALDI-TOF/TOF fragment spectra. p_n_ means proline-directed ions. FA: CH_3_-(CH_2_)_12_-CHOH-CH_2_-CO-.

The results obtained for *B. atrophaeus* are exhibited in Figures 4A–F and compared with those determined for the type strain *B. atrophaeus* DSM 7264^T^. Two test strains DSM 32285 and DSM 29418 were investigated. Figures 4A,B show the MALDI-TOF mass spectra of surface extracts from these bacteria. In contrast to *B. subtilis*, the *B. atrophaeus* strains produce iturin compounds (Bonmatin et al., 2003; Vater et al., 2003). DSM 32285 produces C16-C19 iturins C (Vater et al., 2002, 2003), while DSM 29418 forms C15–C17 mycosubtilins (Bonmatin et al., 2003), which dominate strongly in the mass spectra. In addition, minor amounts of surfactins and fengycins were found at the surface of these organisms. Remarkably, surface extracts of DSM 29418 contain a product with high intensity at *m/z* = 2754.6 which still needs to be identified. This metabolite is specific for *B. atrophaeus.* DSM 32285 revealed a similar product at *m/z* = 2783.5, but with much lower intensity. In Figures 4C,D mass spectra of the culture supernatants of these strains are presented. Also here the iturin compounds dominate, but the fengycin peaks appeared with appreciably higher intensities than in the surface extracts. Obviously, a major part of the fengycins was released into the culture medium. In both spectra the siderophore bacillibactin was visible ([M + H,Na,K]^+^ = 883.4; 905.4 and 921.4). The highest intensity was found for its sodium adduct at *m/z* = 905.4. The product at *m/z* = 2754.6 appeared also in the culture supernatant of strain DSM 29418 in high amount. Figure 4E shows the mass spectra of the surfactin and iturin C clusters produced by DSM 32285, while the mycosubtilin family formed by DSM 29418 is exhibited in Figure 4F. The product patterns of these strains were compared with that of the *B. atrophaeus* type strain DSM 7264^T^. Its products essentially correspond to those found for strain DSM 29418 (surfactin; mycosubtilin; fengycin and the compound with *m/z* = 2754.6), but a characteristic feature of the type strain is the high amount of fengycins released into the culture medium. The mass spectrometric sequence determination of a C16-fengycin A by LIFT-MALDI-TOF/TOF MS is shown in Figure 5. In the lower part of this figure the immonium ions for the amino acid components and the mass peaks for the di- and tripeptide fragments are listed. In the upper part, the complete sequence determination of the C16-fengycin is demonstrated for a ring opening between Tyr(3) and a-Thr(4). In addition, a partial sequence segment ranging from Pro(7) via the lactone linkage between Ile(10) and Tyr(3) to Ala(6) is shown. It corresponds to the sequence of the peptide ring with loss of the side chain attached to L-Tyr (3). Fengycin contains proline in position 7. In the mass spectrometric fragmentation of such compounds the peptide ring preferentially is cleaved N-terminally of proline, frequently with the consequence that proline-directed product ions p_n_ dominate in the fragment spectra.

The structures of mycosubtilin and iturin C produced by the test strains DSM 32285 and DSM 29418 were also determined by LIFT-MALDI-TOF/TOF fragment analysis. In Supplementary Figure S3 the fragment pattern of C17-iturin C produced by strain DSM 32285 is demonstrated with a parent ion [M + H]^+^ = 1084.6. Proline-directed p_n_ and y_n_ – product ions were used for mass spectrometric sequence determination. The fragment pattern for structure elucidation of C15-mycosubtilin formed by *B. atrophaeus* DSM 29418 with parent ions [M + H,Na]^+^ = 1057.6 and 1079.6 is shown in Supplementary Figure S4.

### Subcomplex b: *B. amyloliquefaciens* and *B. velezensis*

**FIGURE 6:**
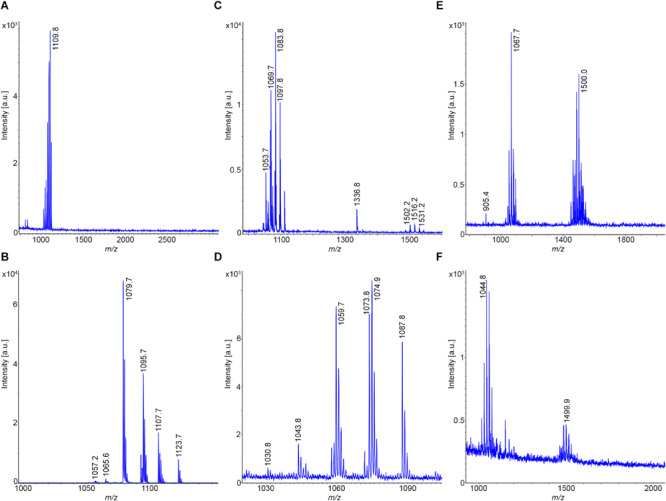
Detection of bioactive peptides produced by *B. amyloliquefaciens*, subsp. *amyloliquefaciens* DSM 7^T^ (type strain) and DSM 32875 as well as *B. velezensis* DSM 23117^T^ (FZB42) and DSM 29399 (FZB45). **(A,B)** MALDI-TOF mass spectra of surface extracts of *B. amyloliquefaciens* DSM 7^T^ and DSM 32875 grown on Landy-agar. **(C,D)** MALDI-TOF mass spectra of surface extracts of *B. velezensis* DSM 23117^T^ (FZB42; type strain) and DSM 29399 (FZB45) grown on Landy-agar. **(E,F)** MALDI-TOF mass spectra of culture supernatants of these strains cultivated for 24 h at 30°C in the Landy medium.

Figure 6A shows the MALDI-TOF mass spectrum of a surface extract of the type strain DSM 7^T^ of *B. amyloliquefaciens*, subsp. a*myloliquefaciens*, which was grown on Landy-agar for 24 h at 30°C. The dominating lipopeptide product is iturin A. C14-C16-iturin A variants were detected. Surfactins were found at a lower amount, while fengycins were completely missing. Similar results were obtained for strain DSM 32875 (see Figure 6B), which clearly belongs to same subspecies as DSM 7^T^. Here C14-C17 iturins A were found.

**FIGURE 7:**
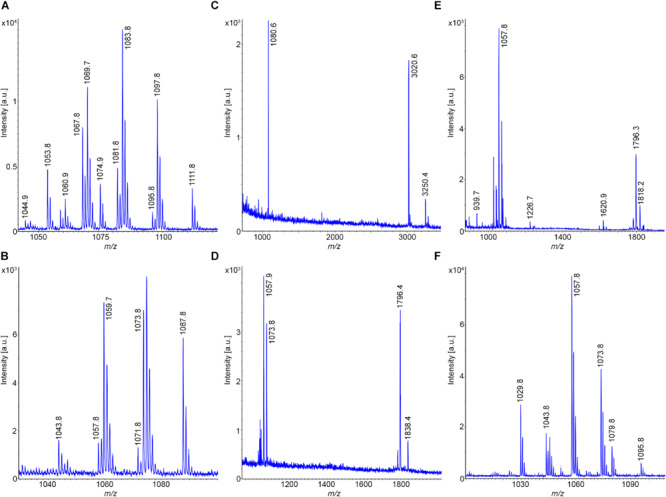
Detection of bioactive peptides produced by *B. velezensis* and *B. licheniformis*. **(A,B)** MALDI-TOF mass spectra of the bacillomycin D and L complexes produced by strains DSM 23117^T^ (FZB42) and DSM 29399 (FZB45), respectively. C14-C17 bacillomycins D and C14-C16 bacillomycins L were detected. **(C–E)** MALDI-TOF mass spectra of surface extracts of *B. licheniformis* DSM 13^T^ (type strain) **(C)**; DSM 32874 **(D);** and M18 **(E)**. **(F)** MALDI-TOF mass spectrum of the lichenysin-complex produced by strain DSM 32874. C13–C15 lichenysins were detected.

In contrast, the type strain for *B. amyloliquefaciens*, subsp. *plantarum*, *B. velezensis* DSM 23117^T^ (FZB42) exhibits a particularly rich product pattern (Chen et al., 2007; Fan et al., 2018). A mass spectrum of this bacterium, which was grown under the same conditions as DSM 7^T^, is shown in Figure 6C. It produces all three families of lipopeptides, the surfactins, bacillomycins D as iturin compounds and fengycins, together with the TOMM microcin plantazolicin. Bacillomycins D were the main compounds in the surface extract. In Figure 6E the mass spectrum of a sample of the culture supernatant of DSM 23117^T^ (FZB42) is shown, which was cultivated in the Landy medium for 24 h at 30°C. Here bacillibactin appeared. Again, the intensity of the fengycin mass peaks was higher than in the mass spectra of the surface extracts, which was also observed for the tested *B. atrophaeus* strains. Similar results have been obtained for strains Rs-MS87 and Sc-K143, which can be classified as *B. velezensis*. A variation was observed for DSM 29399 (FZB45), which has also been identified as a *B. velezensis* strain. In contrast to the type strain DSM 23117 (FZB42), it produces bacillomycin L instead of the D-variant as main product (see Figures 6D,F). Additionally, unlike the other examined strains here plantazolicin is completely missing. The MALDI-TOF mass spectra of the bacillomycin D and L- complexes are displayed in Figures 7A,B. In the surface extracts of strains FZB42 (DSM 23117^T^), Rs-MS87 and Sc-K143 C14-C17 bacillomycins D were detected. DSM 29399 (FZB45) produced C14–C16 bacillomycins L. Mass spectrometric structure analysis was performed for C14-bacillomycin D (parent ions for the protonated and sodiated forms: m/z = 1031.5 and 1053.5) from FZB42 and for C14-bacillomycin L (parent ions for the protonated and sodiated forms: m/z = 1021.5 and 1043.5) from FZB45 by LIFT-MALDI-TOF/TOF fragment analysis. The corresponding fragment patterns for their sequence determination are shown in Supplementary Figures S5, S6.

### Subcomplex c: *B. licheniformis*

Two *B. licheniformis* strains, DSM 32874 and M18, were investigated for their production of bioactive peptides and compared with the data obtained for the *B. licheniformis* type strain DSM 13^T^. MALDI-TOF mass spectra of surface extracts of DSM13^T^; DSM32874 and M18 are shown in Figures 7C–E. The test strains DSM 32874 and M18 produced lichenysins which are lead metabolites of *B. licheniformis* (Pecci et al., 2010*;* Madslien et al., 2013; Favaro et al., 2016). The type strain DSM13^T^, however, is deficient in lichenysin production. Here, such compounds were neither found at the surface of this organism nor were they released into the culture medium. Lichenysins are closely related to surfactins by amino acid replacements. Frequently, L-Glu in position 1 of the surfactin sequence is mutated to L-Gln with the consequence that the mass numbers of lichenysins are by one mass lower than the corresponding surfactins. The lichenysin complex produced by DSM 32874 is exhibited in Figure 7F. C13–C15-lichenysins were detected.

Another characteristic product of *B. licheniformis* is lichenicidin (Begley et al., 2009; Dischinger et al., 2009; Caetano et al., 2011), a two component lantibiotic with mass numbers of *m/z* = 3020.6 and 3250.4, respectively. These lantipeptides specifically were found to be attached to the surface of such bacteria. They were not released into the culture medium. Lichenicidins dominate in the mass spectra of surface extracts of the type strain DSM 13^T^, which is a potent producer of these lantibiotics. They were visible in a growth period between 12 and 30 h. In the corresponding mass spectra of the test strains DSM 32874 and M18 they were found in only rather low amount.

Interestingly, in the mass spectra of the tested *B. licheniformis* strains several not yet identified compounds were detected. A prominent mass peak was found for all investigated test strains at *m/z* = 1796.4. This mass number refers to the protonated form of this compound. In addition, also peaks for its alkali adducts were monitored together with mass peaks at *m/z* = 1838.4 and 1880.2 which compared with the main species show a difference of 42 and 84 mass units, respectively. Presumably, these peaks represent the mono- and bi-acetylated forms of this compound. In the mass spectra of the type strain DSM 13^T^ which is lacking m/z = 1796.4 another compound was found with a mass number of *m/z* = 1080.6 that remains to be identified. Lichenicidins were selectively found in surface extracts, when DSM 13^T^ was grown on TSA-agar, while the compound with *m/z* = 1080.6 appeared specifically in samples cultivated on Landy-agar.

MALDI-TOF mass spectra of the culture supernatants of DSM 32874 and M18 also revealed the presence of lichenysins and the compound at *m/z* = 1796.4. The structure of a C15-lichenysin (parent ions for the protonated and sodiated forms: *m/z* = 1035.7 and 1057.4) from strain DSM 32874 was determined by LIFT-MALDI-TOF/TOF fragment analysis as shown in Supplementary Figure S7.

### Subcomplex d: *B. pumilus*

**FIGURE 8:**
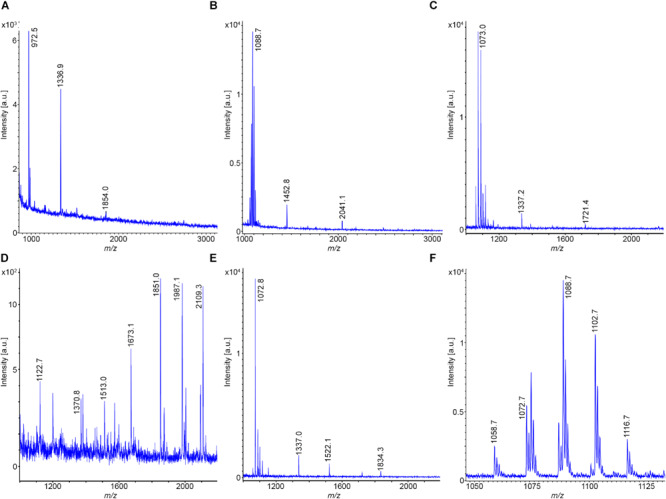
Detection of bioactive peptides produced by *B. pumilus.* **(A–C)** MALDI-TOF mass spectra of surface extracts of *B. pumilus* DSM 27^T^ (type strain) **(A)**; Abi11 **(B)** and Rs-Ts276 **(C)**. MALDI-TOF mass spectra of the culture supernatant of *B. pumilus* Rs-Ts276 grown in the Landy-medium for 24 h at 30°C **(D)** and of a surface extract of the cell pellet obtained after 72 h **(E)**. **(F)** MALDI-TOF mass spectrum of the pumilacidin-complex produced by Abi11. C15–C19 pumilacidins were detected.

Two *B. pumilus* strains, Abi11 and strain Rs-Ts276, were investigated for their biosynthetic potential and compared with that of the type strain DSM 27^T^. In Figures 8A–C MALDI-TOF, mass spectra of surface extracts of these strains are displayed. The test strains Abi11 and Rs-Ts276 produce pumilacidins, which are also closely related to surfactins. Pumilacidins (Naruse et al., 1990), prominent lipopeptides of *B. pumilus*, differ from surfactins by longer fatty acid side chains (C13-C15–ß-OH-fatty acids for surfactins and C15-C19 – species for pumilacidins).

Remarkably, the type strain DSM 27^T^ is deficient in pumilacidin production, while the test strains Abi11 and Rs-Ts 276 are good pumilacidin producers. Obviously, these lipopeptides are attached to the outer cell wall. Therefore, they were predominantly found in surface extracts of *B. pumilus* cells grown on agar plates or were obtained from cell pellets sedimented from liquid cultures. On the other hand, the yield of pumilacidins was low in culture filtrates. They were found over the complete range of cultivation times from 10 to 72 h.

Similar to *B. subtilis* and *B. velezensis*, most of the *B. pumilus* strains form plantazolicin, which specifically was found in surface extracts. For example, good yields of this compound was obtained from DSM 27^T^ cells picked from LB and TSA- agar plates (Figure 8A). Plantazolicin was also formed by Rs-Ts 276 (Figure 8C), but not by Abi 11 (Figure 8B). It was not observed in culture filtrates of the investigated *B. pumilus* strains. Strain Rs-Ts276 produces compounds with mass numbers of m/z = 1851.0; 1987.1; and 2109.3 which have still to be identified (Figure 8D). They appeared in the growth period between 8 and 24 h and were released into the culture medium. They dominate in MALDI-TOF mass spectra, when TSA is used as growth medium. They were not found in surface extracts of cell pellets sedimented by centrifugation from liquid cultures (Figure 8E). Figure 8F shows a MALDI-TOF spectrum of the pumilacidin-complex produced by *B. pumilus* Abi 11. C15-C19 pumilacidins were detected.

### Profiling of the Investigated PGPR-Strains for Volatiles

The volatilomes of the selected test- and type strains have been investigated. Volatiles spectra were established by using HS SPME GC-MS, compared to the NIST MS database 14 and confirmed via Kovats indices. Organic bacterial volatiles can be clustered in six different groups: hydrocarbons, ketones and alcohols, acids, sulfur and nitrogen-containing compounds and terpenes (Audrain et al., 2015; Kanchiswamy et al., 2015). Volatile profiles frequently are highly overlapping and difficult to detect due to their low concentration. The investigated rhizobacteria revealed mainly volatile hydrocarbons (alkanes and alkenes), alcohols, ketones, few sulfur-containing compounds and nitrogen-containing pyrazines. The number of volatiles produced by these isolates range from four up to seventeen (Table 3).

**TABLE 3:** Volatile organic compounds produced by different members of the *Bacillus subtilis* species complex. Identification was performed via HS-SPME GC-MS, matching to the NIST Mass Spectral Database and confirmation by the Kovats-Index.

	***Bacillus subtilis***		***Bacillus atrophaeus***			***Bacillus amyloliquefaciens***		***Bacillus***			***Bacillus licheniformis***			***Bacillus pumilus***					
**Compound**	**Kovats-Index**	**DSM 32873**	**Sc-S7**	**DSM 7264**	**DSM 29418**	**DSM 32285**	**DSM 7**	**DSM 32875**	**DSM 23117**	**DSM 29399**	**Rs-MS87**	**DSM 13**	**DSM 32874**	**M 18**	**DSM 27**	**Abi11**	**Rs-Ts276**	**Biological function**	**References**
Methanethiol	401			x			x					×			×			Discoloration of *Agaricus bisporus*. *Pleurotus ostreatus* mycel inhibition	Lo Cantore et al., 2015
Bicyclo[2.1.0] pentane	508							×		×								n.a.	
Isoprene	510	×		×							×				×	×		Protection against heat stress, stabilizes cell membranes in response to heat stress; bacterial interactions	Wilkins, 1996; Tyc et al., 2017
1,4-Pentadiene	510	×	×		×	×		×	×	×		×	×	×	×	×	×	n.a.	
1-Propanol	561			×											×			n.a.	
2-Methylfuran	608																×	Contributes to bacterial interactions	Tyc et al., 2017
2-Methyl-1-propanol*	618											×						n.a.	
2,3,4-Trimethyloxetane	655	×	×	×	×	×						×	×	×		×		n.a.	
1-Chlorobutane	656						×	×									×	n.a.	
1-Butanol	659														×			n.a.	
2,3-Butanedione	684						×				×	×						n.a.	
2-Pentanone	684										×							n.a.	
Acetoin	708							×	×	×	×						×	Plant growth promotion and ISR activation in *A. thaliana*, increase of root length and number of lateral roots in *Lactuca sativa*	Ryu et al., 2003, 2004; Rudrappa et al., 2010; Fincheira et al., 2016
3-Methyl- butanenitrile	718											×						n.a.	
3-Methyl-1-butanol	725						×				×	×	×	×			×	Growth promotion and enhanced salinity tolerance of *A. thaliana*	Ledger et al., 2016
1-Chloropentane	725												×	×			×	n.a.	
2-Methyl-1-butanol	728											×		×			×	Inhibtion of appressoria germination and germination of *Phyllosticta citricarpa*	Toffano et al., 2017
3,4-epoxy-2-pentanone*	728															×		n.a.	
Dimethyl sulfone	733	×	×		×	×		×	×	×				×			×	n.a.	
Dimethyl disulfide	733	×	×		×	×	×		×	×	×	×		×				Increase of plant biomass of *A. thaliana*, reduces mycelium growth and sclerotia germination of *S. sclerotiorum*, broccoli growth increase	Groenhagen et al., 2013; Giorgio et al., 2015; Lo Cantore et al., 2015
3-Methyl-2-pentanone	741				×	×	×	×	×	×	×					×	×	n.a.	
3-Metho×y-3-methyl-2-butanone	758						×		×	×	×		×	×		×	×	n.a.	
3,3-Dietho×y-1-propyne*	784						×					×						n.a.	
Butyl acetate	802																	n.a.	
Acetone	847	×	×		×		×	×		×	×	×	×				×	n.a.	
2-Methylbutanoic acid	848						×										×	Attract *Episyrphus balteatus*; induced *E. balteatus* oviposition	Leroy et al., 2011
2-Heptanone	881										×							n.a.	
1-Isothiocyanato-2-methylpropane*	942											×						n.a.	
6-Methyl-2-heptanone	948				×	×			×	×	×					×	×	n.a.	
5-Methyl-2-heptanone	958										×						×	n.a.	
2-Ethyl-1-Hexanol	1019						×					×						Mycel growth inhibition and reduction of sclerotial germination of *S. sclerotiorum*	Fernando et al., 2005
2-Isopropyl-5-methylpyrazine	1039																×	n.a.	
2-Decanone	1154									×								Inhibition of mycelial growth of *R. solani*	Weise et al., 2012
2,3,5-Trimethyl-6-propylpyrazine*	1183																×	n.a.	
2,3-Diethyl-5-methylpyrazine*	1264																×	n.a.	

*Assignment as mVOCs refers to the absence in the mVOC database (Lemfack et al., 2018).

Similar to the bioactive peptides the members of the *B. subtilis* complex can be classified according to their volatile production. Some of the detected VOCs, such as hydrocarbons, like 1,4 pentadiene and isoprene or sulfur containing compounds, like dimethyl sulfone and dimethyl disulfide were found wide-spread among the investigated strains, but other classes of VOCs can be specifically attributed to definite subcomplexes. For, example, acetoine was specifically produced by *B. amyloliquefaciens* and *B. velezensis* strains. 2,3,4-trimethyl-methoxetane is characteristic for *B. subtilis*, *B. atrophaeus* and *B. licheniformis* strains. The production of keto-compounds, such as 3-methyl-2-pentanone, 5- and 6-methyl-2-heptanone or 3-methoxy-3-methyl-2-butanone was observed for *B. atrophaeus, B. amyloliquefaciens, B. velezensis* and *B. pumilus* strains. While especially organisms belonging to *B. velezensis* (*B. amyloliquefaciens* ssp. *plantarum*) revealed a high variety of produced ketones, 5-methyl-2-heptanone and 6-methyl-2-heptanone could not be identified as volatile secondary metabolites of *B. subtilis* and *B. licheniformis* strains. 3-Methoxy-3-methyl-2-butanone was found in isolates belonging to the *B. amyloliquefaciens* subcomplex as well as to *B. licheniformis* and *B. pumilus* strains, but not for *B. subtilis* and *B. atrophaeus*. Alcohol-compounds, such as 2-methyl-1-propanol or 2- and 3-methyl- 1-butanol were predominantly formed by *B. licheniformis*. Alcohols, contributing with 16% to the overall diversity of microbial volatiles (Schenkel et al., 2015), did not appear in the volatile spectra of *B. subtilis* and *B. atrophaeus*. Dimethyl disulfide, reported for both growth promotion and reduction in *Arabidopsis thaliana* (Kai et al., 2010; Meldau et al., 2013), was not observed in the chromatograms of *B. pumilus*. Chlorobutane was specifically exhibited by *B. amyloliquefaciens*, subsp. *amyloliquefaciens*, whereas chloropentane was produced by *B. licheniformis*. The tested *B. pumilus* strains show a relatively high variety in volatile production. In particular, *B. pumilus* Rs-Ts276 is distinguished by an exceptional pattern of VOC-production, comprising hydrocarbons, alcohols, ketones as well as chlorobutane and chloropentane. A specific feature of this organism is the occurrence of pyrazine compounds. Alkylpyrazines show antifungal activity and inhibit sclerotia germination ability (Haidar et al., 2016; Mülner et al., 2019).

Furthermore, several mVOCs appeared in the volatilome of only one strain: 2-methylfuran in *B. pumilus* Rs-Ts276, 3-methylbutanenitrile and 1-isothiocyanato-2-methylpropane in *B. licheniformis* DSM13^T^, 2-pentanone in *B. velezensis* Rs-MS87, 1-butanol in *B. pumilus* DSM27^T^ and 3,4-epoxy-2-pentanone in *B. pumilus* Abi11. Moreover, there was not a single volatile compound that could be detected in all investigated members of the *B. subtilis* species complex. The most abundant substance revealed to be 1,4-pentadiene that was identified in all strains apart from Rs-MS87, DSM 7264^T^, DSM 7^T^, and DSM 27^T^.

### Field Trial Experiments With the Investigated PGPR Organisms

**FIGURE 9:**
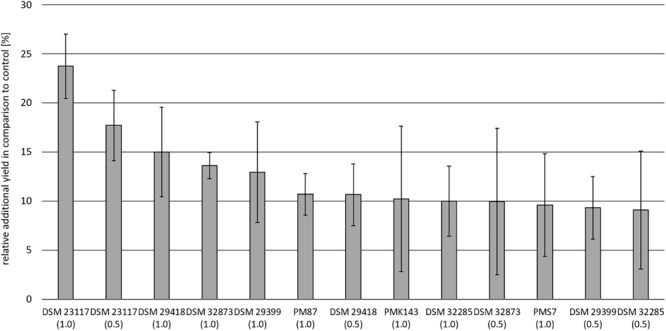
Field trial experiments with the investigated PGPR-strains of the *B. subtilis* complex with potato as target plant. Difference annual yield over control (without treatment with a PGPR strain) in percent demonstrating the growth stimulating effects of the investigated rhizobacteria. 0.5, l, or 1 l spore suspension of the PGPR strains in 300 l water were applied per hectare. Cultivar: “Goldmarie”; soil-type: slightly loamy sand; plot = 21 m^2^. The data are the mean of four randomized repetitions. Bars indicate standard errors between the replicates.

**FIGURE 10:**
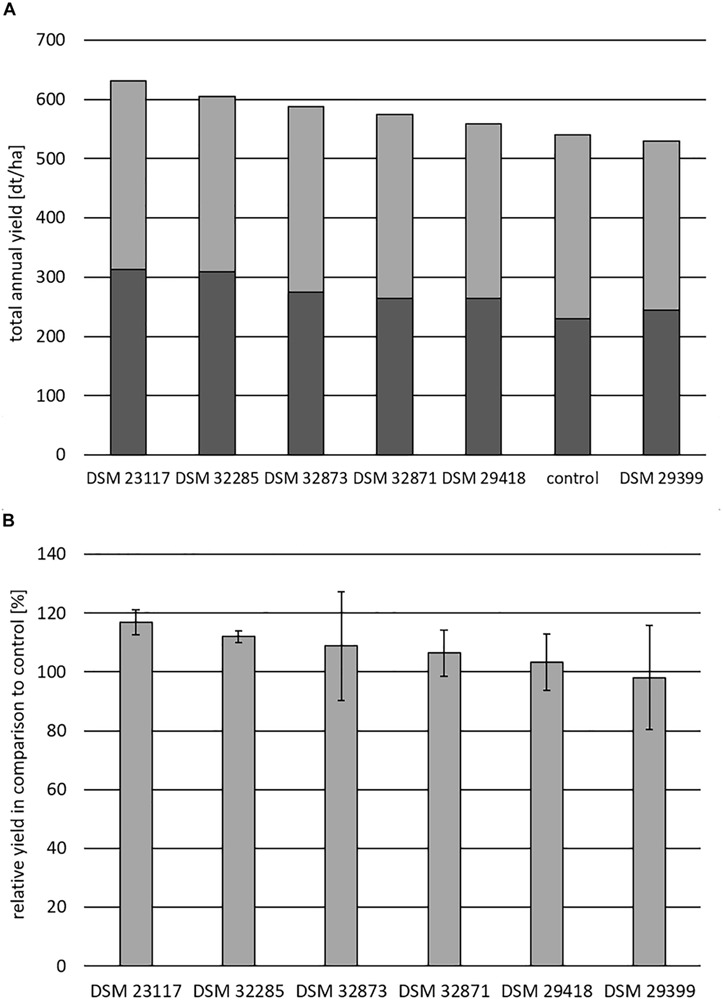
Field trial experiments with the investigated PGPR-strains of the *B. subtilis* complex with maize as target plant. **(A)** Shows the total annual yield in dt/ha obtained in the presence of the investigated rhizobacteria in comparison to the control without PGPR strains. The yield is differentiated in plant biomass (gray) and ear (black). **(B)** The relative yield in percent in the presence of the investigated PGPR strains compared with the control is exhibited. Cultivar: “Colisee”; soil-type: slightly loamy sand; plot = 18 m^2^. The data are the mean of four randomized repetitions. Bars indicate standard errors between the replicates.

To evaluate their practical usage field trial experiments were performed with the investigated PGPR organisms using potato and maize as target plants. The achieved results were presented in Figures 9, 10. The relative additional yield in comparison to control in percent for potato is demonstrated for all tested rhizobacteria in Figure 9. The highest growth stimulation of approx. 25% was obtained for DSM 23117^T^ (FZB42), the type strain for *B. velezensis*. Good growth promotion was also achieved by *B. atrophaeus* DSM 29418; *B. subtilis* DSM 32873 and *B. velezensis* DSM 29399 (FZB45), while the other organisms showed growth stimulation effects of at least 10%. In general, an increased concentration (1 l spore suspension) of the PGPR strain led to an increase in potato yield, which is especially visible for DSM 23117^T^ (FZB42).

The results for the maize field trials are presented in Figures 10A,B. Figure 10A shows the total annual yield in dt/ha. The control in the absence of the tested rhizobacteria amounted to approximately 530 dt/ha, dividing into approximately 200 dt/ha for the plant biomass and approximately 300 dt/ha for the ears. The relative yield in comparison to control in percent for maize is exhibited in Figure 10B. The production of the control was set to 100%. Again, *B. velezensis* DSM 23117^T^ (FZB42) showed the highest growth stimulation effect of approximately 90 dt/ha (approximately 17%) followed by *B. atrophaeus* DSM 32285 (12%) and *B. subtilis* DSM 32873 (8.5%).

## Discussion

The members of the *B. subtilis* group are of high importance as industrially relevant *Bacillus* strains. Recent applications are focused on the enhancement of crop productivity and disease resistance as biostimulants and biopesticides. Most of the characterized members of the *B. subtilis* complex are PGPR. Some of them, as for example DSM 23117^T^ (FZB42), the type strain of *B. velezensis*, are commercially applied to increase the productivity of numerous useful plants. The exploitable properties of these strains are essentially conferred by a wide spectrum of metabolites that are naturally produced by these microorganisms. Of particular importance for the activities and industrial utilization of such strains is their equipment with bioactive peptides and volatiles. In this study we performed a comprehensive screening of 13 industrially relevant test strains of the *B. subtilis* group for the production of such compounds, which are of high importance for their abilities as biofertilizers and biocontrol agents. For the rapid, reliable and sensitive detection of such compounds, mass spectrometric techniques are the tools of choice. In particular, MALDI-TOF MS is excellently suited to monitor metabolites, such as (lipo)peptides, polyketides and hybrids thereof, lantibiotics and biocines directly in cellular extracts and culture supernatants. On the other hand volatiles can be efficiently analyzed by GC-MS. Using such techniques, we identified and characterized the test organisms and determined their profiles for bioactive peptides and volatiles.

**FIGURE 11:**
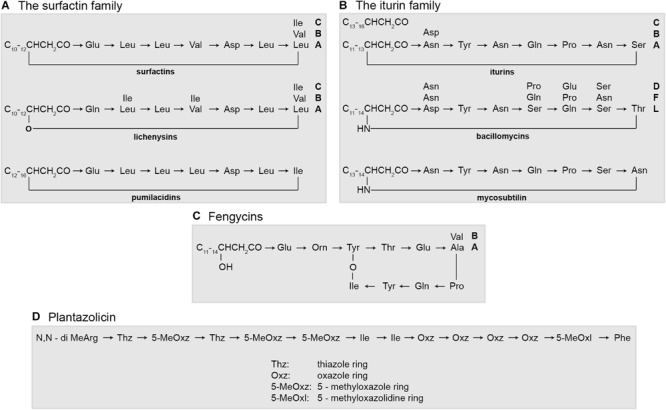
The structure of bioactive peptides produced by members of the *B. subtilis* complex.

In the first step, the test organisms were identified and attributed to the four subcomplexes of the *B. subtilis* complex by MALDI-TOF MS. Thereafter, we determined their profiles for the production of bioactive peptides and volatiles. The obtained results were summarized in Tables 1, 3. They demonstrate that each subgroup is distinguished by specific sets of such compounds. As far as bioactive peptides are concerned the following features can be derived:

The members of all subcomplexes ubiquitously produce surfactins or related lipopeptides, such as lichenysins (*B. licheniformis*) and pumilacidins (*B. pumilus*) as well as the siderophore bacillibactin. Iturin compounds (iturins A and C; bacillomycins D, F and L; mycosubtilins) were produced specifically by *B. atrophaeus*, *B. amyloliquefaciens*, and *B. velezensis*, but not by *B. subtilis*, *B. licheniformis*, and *B. pumilus*. Plantazolicin is formed by *B. subtilis*, *B. velezensis*, and *B. pumilus*. Fengycin, an antifungal lipotridecapeptide and efficient biocontrol agent against filamentous fungi, was found for *B. subtilis, B. atrophaeus, B. amyloliquefaciens*, and *B. velezensis*, but not for *B. licheniformis* and *B. pumilus*. The structure of all these bioactive compounds is demonstrated in Figure 11.

Combining these features with specific biomarkers of members of the different subgroups of the *B. subtilis* complex (see Table 2) enables a rapid and reliable mass spectrometric identification of organisms that belong to the *B. subtilis* group without the need to consult an expensive database. For this procedure merely a disintegration mass spectrum of a strain has to be combined with a MALDI-TOF mass spectrum of a surface extract of this organism, which both can be afforded in a minimum of time. This approach yields specific information about cellular components and bioactive products, which can be used to identify an unknown bacterial strain. Results obtained with this simple approach assorts well with the identification on the basis of data base evaluation.

The profiles for bioactive peptides derived from mass spectrometric evaluation of the test strains essentially are compatible with the wealth of knowledge obtained on the genetic level from whole genome sequencing (Fan et al., 2017; Harwood et al., 2018; Dunlap et al., 2019) Table 4 shows the presence of the corresponding gene clusters in the genomes for the production of the bioactive peptides of the investigated PGPR-strains. For the detection of the gene clusters the complete genome sequences from the test strains Sc-S7, Rs-MS87 andSc-K143 as well as the type strains 168^T^, DSM7^T^, DSM13^T^, and DSM27^T^ were available. For these organisms the gene cluster information was obtained by antiSMASH-4.0-analysis (Blin et al., 2017). For the other strains the relevant gene clusters were derived from the detected products.

**TABLE 4:** Gene clusters in the genomes for bioactive peptides of the investigated PGPR-strains.

**Strain**	**Classification by MALDI-TOF MS**	**Gene clusters**
**Subcomplex a: *B. subtilis* and B. *atrophaeus***		
168^T^	*B. subtilis*	*srf, fen*, deficient *sfp*-gene
DSM 32873	*B. subtilis*	*srf, fen, pzn*
Sc-S7	*B. subtilis*	*srf, fen*
DSM 7264^T^	*B. atrophaeus*	*srf, myc, fen*
DSM 32285	*B. atrophaeus*	*srf, ituC, fen*
DSM 29418	*B. atrophaeus*	*srf, myc, fen*
**Subcomplex b: *B. amyloliquefaciens* and *B. velezensis***		
DSM 7^T^	*B. amyloliquefaciens*	*srf, ituA, fen*D-E, deficient in *fen*A-C
DSM 32875	*B. amyloliquefaciens*	*srf, ituA*
DSM 23117^T^ (FZB 42)	*B. velezensis*	*srf, bmyD, fen, pzn*
DSM 29399 (FZB 45)	*B. velezensis*	*srf, bmyL, fen*
Rs-MS87	*B. velezensis*	*srf, bmyD, fen, pzn*
Sc-K143	*B. velezensis*	*srf, bmyD, feng, pzn*
**Subcomplex c: *B. licheniformis***		
DSM 13^T^	*B. licheniformis*	lch, licA1, licA2
DSM 32874	*B. licheniformis*	lch
M18	*B. licheniformis*	lch
**Subcomplex d: *B. pumilus***		
DSM 27^T^	*B. pumilus*	*pml, pzn*
Abi 11	*B. pumilus*	*pml, pzn*
Rs-Ts276	*B. pumilus*	*pml, pzn*

*Srf, fen, pzn, myc, ituA, bmyD, bmyL, lch, licA1, *lic*A2 and pml indicate the gene clusters for the production of surfactin, fengycin, plantazolicin, iturin A, bacillomycin D, bacillomycin L, lichenysin, lichenicidin A1 and A2 and pumilacidin, resp. The gene cluster were detected either by antiSMASH 4.0 analysis of the genome sequences for the type strains and for strains Sc-S7, Rs-M87, Sc-K143 or on the basis of the bioactive peptides produced by the other strains for which genome sequences are not yet available.*

Mass spectrometric and chemical analysis are the ultimate criteria which metabolites are really produced. This situation is particularly evident from the comparison of the production of bioactive peptides by the investigated type- and test-strains. While the test strains frequently exhibited a widely complete product spectrum, most of the type strains revealed deficiencies concerning the production of essential metabolites. The most prominent example is the type strain 168^T^ for *B. subtilis*, which does not produce any non-ribosomally formed peptide or polyketide. Though the biosynthetic genes for the biosynthesis of surfactin and fengycin, for example, were found intact in the genome of this strain, both lipopeptides were not produced because of a deficient Sfp-protein preventing the loading of the 4′-phosphopantetheinyl moiety to the T-domains of the corresponding synthetases (Nakano et al., 1992; Cosmina et al., 1993; Kunst et al., 1997). DSM 7^T^, the well known type strain for *B. amyloliquefaciens*, does not produce fengycins. In the genome of DSM 7^T^ fengycin biosynthetic genes *fen*A-C are missing, while only *fen*D-E are left (Borriss et al., 2011). Therefore, fengycin production is omitted.

Other candidates are *B. licheniformis* DSM13^T^ and *B. pumilus* DSM 27^T^ which in their genomes harbor the gene clusters for the production of lichenysins (Veith et al., 2004), the lead metabolite of *B. licheniformis*, and pumilacidins, characteristic for the genus *B. pumilus*. Both type strains are deficient in the formation of these compounds which obviously are not expressed. However, DSM13^T^ is a potent producer of the dual lantibiotics lichenicidins (Begley et al., 2009; Dischinger et al., 2009; Caetano et al., 2011), while DSM27^T^ forms plantazolicin.

Subcomplex a of the *B. subtilis* species complex comprises *B. subtilis* and the closely related *B. atrophaeus* strains, which though being closely related show characteristic differences in their product spectra. In contrast to *B. subtilis*, *B. atrophaeus* strains produce iturin compounds, but are lacking plantazolicin. *B. atrophaeus* strains revealed a compound with a mass number of *m/z* = 2754.6 Da, which still has to be identified. This metabolite is specific for the *B. atrophaeus* strains studied.

*Bacillus amyloliquefaciens* (subcomplex b) can be classified into two subspecies *B. amyloliquefaciens*, subspecies *amyloliquefaciens* and *B. amyloliquefaciens*, subspecies *plantarum*, now designated as *B. velezensis* (Fan et al., 2018). *B. velezensis* strains are able to colonize plant roots and organs and to live with plants in a symbiosis as endophytes. These two subspecies can clearly be distinguished mass spectrometrically both as far as their disintegration mass spectrum and their product pattern are concerned. The prototype of subspecies *amyloliquefaciens* is DSM 7^T^, which in contrast to *B. velezensis* strains produces iturins instead of bacillomycins and does not form fengycins and plantazolicin. From all members of the *B. subtilis* complex, *B. velezensis* strains are the most productive organisms as far as secondary metabolites are concerned. A prominent example is the type strain DSM 23117^T^ (FZB42). Approximately 8 kDa of its genome is devoted for the production of such compounds (Chen et al., 2007), among them three lipopeptide families, the surfactins, bacillomycins D and fengycins, the siderophore bacillibactin, plantazolicin, the dipeptide bacilysin and a wide spectrum of volatiles. In addition, it forms amylocyclicin, a ring-shaped biocine with a molecular mass of *m/z* = 6.8 kDa showing antimicrobial activities (Scholz et al., 2014).

*Bacillus licheniformis* strains representing subcomplex c exhibit two specific products, the lichenysins, a lipopeptide family which is related to surfactin and the dual lantibiotics, the lichenicidins. In addition, we detected by MALDI-TOF MS another yet unknown compound with a mass number of *m/z* = 1796.2 Da, which is produced in appreciable extent. It has to be identified by future work.

Subcomplex *d* is formed by *B. pumilus* strains. Here, the main products are pumilacidins, which are also closely related to surfactins sharing longer fatty acid side chains with similar amino acid sequences. *B. pumilus* does not produce iturins and fengycins, but forms plantazolicin.

Microorganisms produce a huge number of low mass volatile organic compounds, which attain increasing attention for agricultural and biotechnological applications. Nowadays, databases are available, which comprise more than 2000 of such substances (Lemfack et al., 2014, 2018). Also, the members of the *B. subtilis* complex produce an extensive pattern of these volatile metabolites which are listed in Table 3. Their nature and action gain increasing attention concerning plant growth promotion (Ryu et al., 2003; Asari et al., 2016; Wu et al., 2018), biocontrol against phytopathogenic bacteria (Rajer et al., 2017; Tahir et al., 2017a; Xie et al., 2018) and fungi (Fernando et al., 2005; Zhang et al., 2013; Chaves-Lopez et al., 2015; Asari et al., 2016; Massawe et al., 2018; Hassan et al., 2019), induction of systemic resistance against phytopathogens (Ryu et al., 2004; Farag et al., 2006, 2013) as well as steering of gene expression and metabolic processes in plants (Tahir et al., 2017b). Similar to bioactive peptides the members of the *B. subtilis* complex show characteristic specificities concerning the produced volatiles. For example, among the different classes of such compounds ketones were frequently produced by *B. atrophaeus, B. amyloliquefaciens, B. velezensis* and *B. pumilus* strains. In particular, acetoine which is well known for its growth promoting and systemic resistance inducing effects (Ryu et al., 2003) could specifically attributed to *B. amyloliquefaciens* and *B. velezensis* strains forming subcomplex 2, while alcohols, such as 2- and 3-methyl-1-butanol were predominantly produced by *B. licheniformis* (subcomplex 3).

The volatilomes, a term first used by Maffei et al. (2011), of the investigated plant growth promoting bacteria were highly overlapping, an effect that is potentially influenced by media, GC MS and the fiber used in the data acquisition process. An omnipresent dilemma in profiling of microbial volatile spectra is that the concentration of many mVOCs is often merely above the threshold. Only two of the so far detected, but not yet described volatiles were unique for one of the investigated bacteria: 3,4-epoxy-2-pentanone in *B. pumilus* Abi11 and 2,3,5-trimethyl-6-propylpyrazine in *B. pumilus* Rs-Ts276. Other bacterial volatiles which were solely measured in one of the investigated strains, were previously characterized in other studies and can be found in the mVOC database (Lemfack et al., 2014, 2018). Overall, 33 volatile substances could be identified including six novel microbial volatiles according to the mVOC database (Table 3). The biological function of the majority of mVOCs has yet to be ascertained. Only 17 out of 40 volatiles have a known function with only three of them associated to plant growth promotion.

All the investigated industrial test strains show promising growth stimulation effects in field trials with potato and maize as model plants demonstrated in Figures 9, 10. The highest effects were always achieved with DSM 23117^T^ (FZB42), the type strain for *B. velezensis* which gains progressive industrial attention. The knowledge of the potential of the investigated strains for the production of bioactive peptides and volatiles presented in this paper will provide the basis for future work to improve their efficacy in plant growth promotion and biocontrol activities to protect plants from infection by phytopathogens.

## Conclusion

Thirteen industrially relevant strains were identified by MALDI-TOF MS as members of the *B. subtilis* community and were attributed to the corresponding subcomplexes. In our work we present a comprehensive mass spectrometric exploration of the bioactive peptides and volatiles produced by the members of the *B. subtilis* complex in a time- and space directed manner for the first time. Each of the four subcomplexes of the *B. subtilis* species complex is equipped with a specific set of such compounds. Their profiles can be used for rapid mass spectrometric typing of their producer organisms. The impact of the tested rhizobacteria on the yield in growing potato and maize was investigated in field trials. Growth stimulation effects between 10 and 25% were obtained. Future research will be focused on the mechanisms of the detected metabolites in plant growth stimulation and plant health improvement. Especially the understanding of the modes of action of mVOCs on plant growth is widely unknown, apart from a few volatiles. Deeper insight and further knowledge will help to identify ideal plant growth promoting bacteria for specific applications in agriculture.

## Data Availability Statement

The original contributions presented in the study are included in the article/Supplementary Material, further inquiries can be directed to the corresponding author.

## Author Contributions

KD, ES, PM, and JV designed and developed the experimental strategy and the concept of the manuscript. ES, PM, KD, and SH did the microbiological work (strain management and sample preparation). JV, SH, and MW performed the generation, evaluation and presentation of the mass spectrometric data. PM studied the volatilomes of the tested strains. KD and HJ initiated and supervised the field trial tests. JV, TC, and PM wrote the manuscript. HJ, PL, TC, and GB were involved in preparing the manuscript. All authors contributed to the article and approved the submitted version.

## Conflict of Interest

PM, ES, KD, HJ, and JV were employed by the company ABiTEP GmbH.

The remaining authors declare that the research was conducted in the absence of any commercial or financial relationships that could be construed as a potential conflict of interest.

## Footnotes

1 http://www.chemspider.com/
