# Supplementary figures and images for: Profiling for Bioactive Peptides and Volatiles of Plant Growth Promoting Strains of the Bacillus subtilis Complex of Industrial Relevance
Source: Front Microbiol. 2020 Jun 30;11:1432. doi: 10.3389/fmicb.2020.01432 (PMC7338577; doi:10.3389/fmicb.2020.01432)

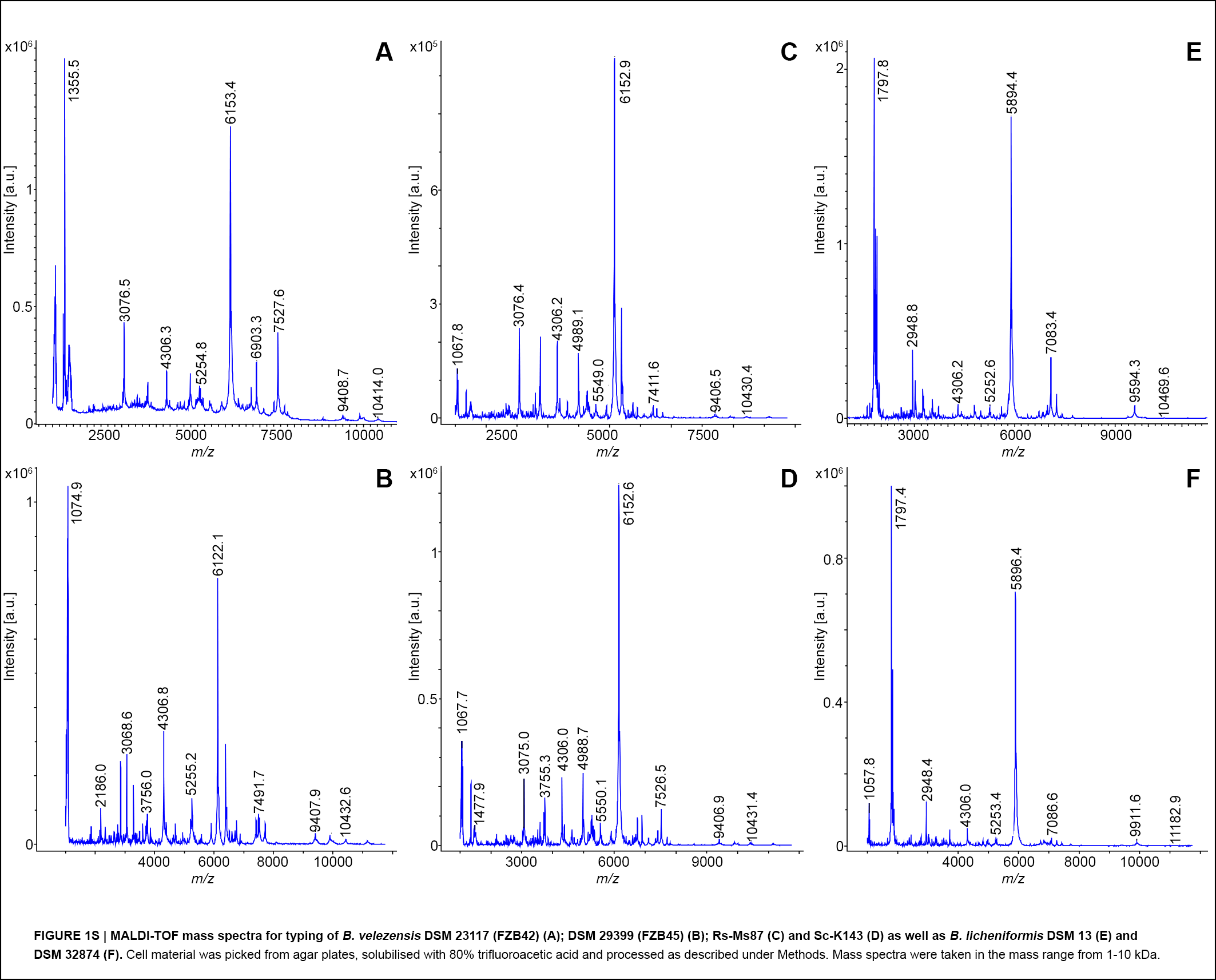

Supplement: Supplementary file 1 [file Image_1.TIF]

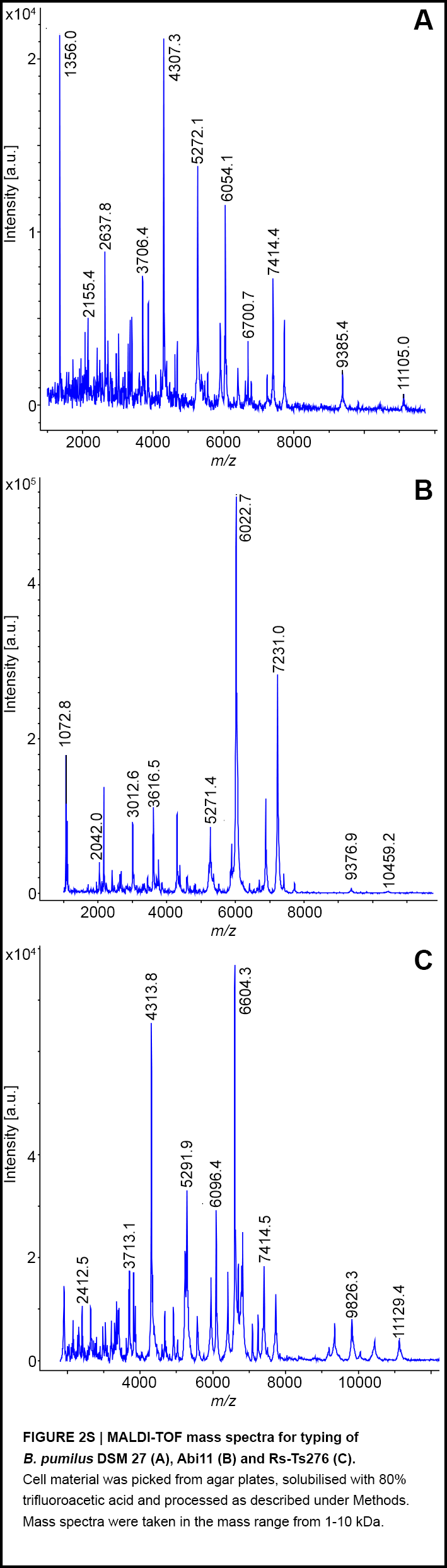

Supplement: Supplementary file 2 [file Image_2.TIF]

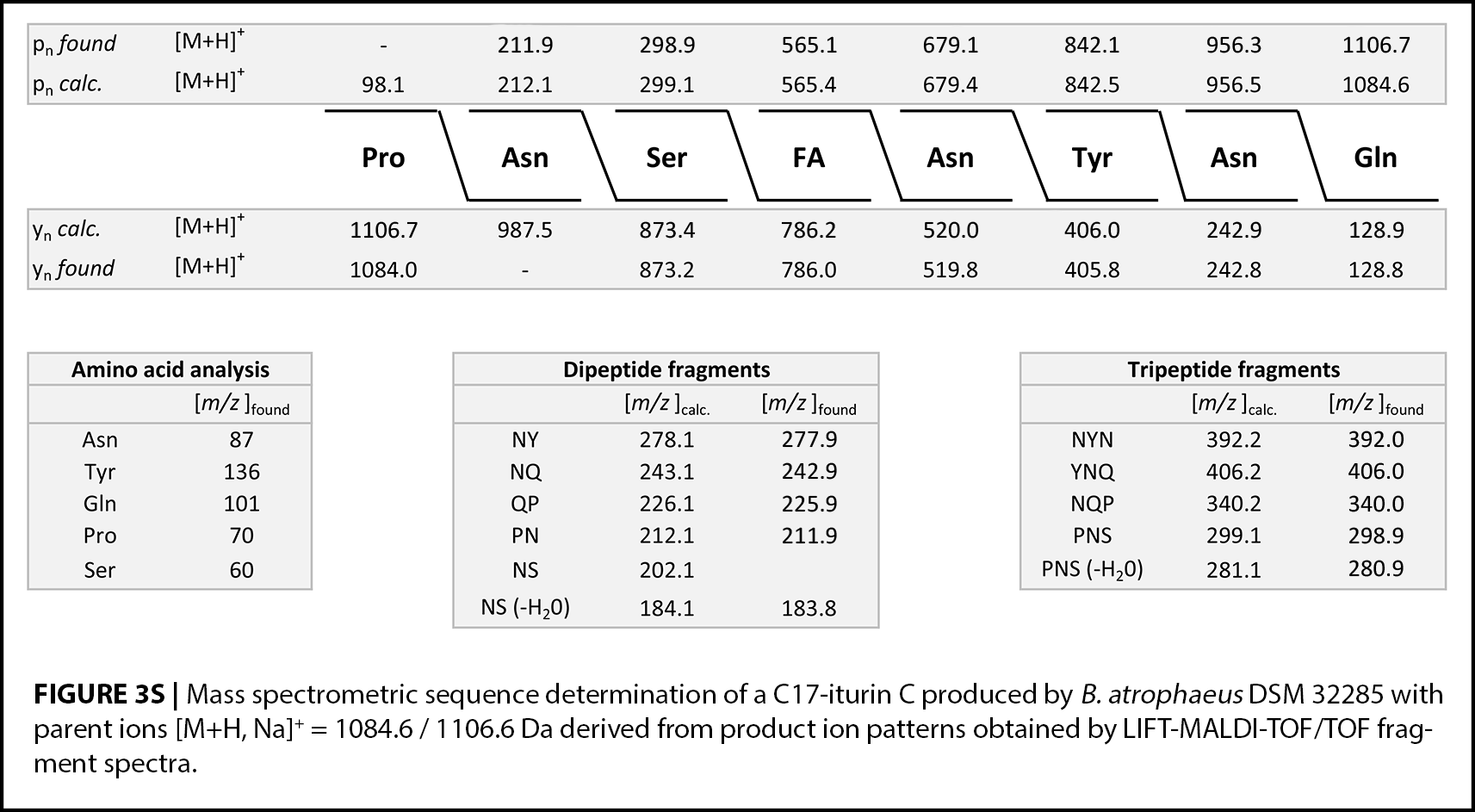

Supplement: Supplementary file 3 [file Image_3.TIF]

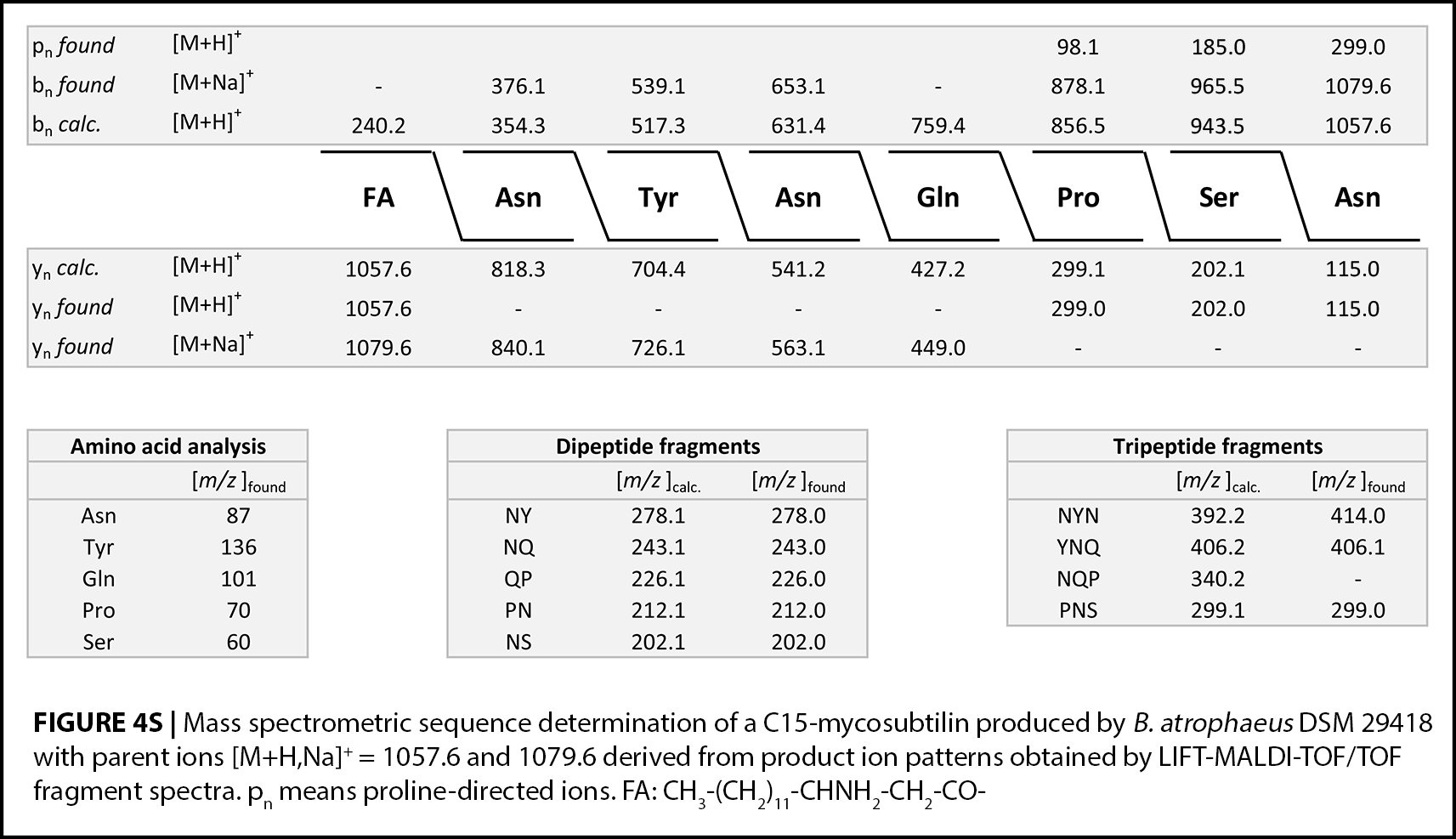

Supplement: Supplementary file 4 [file Image_4.TIF]

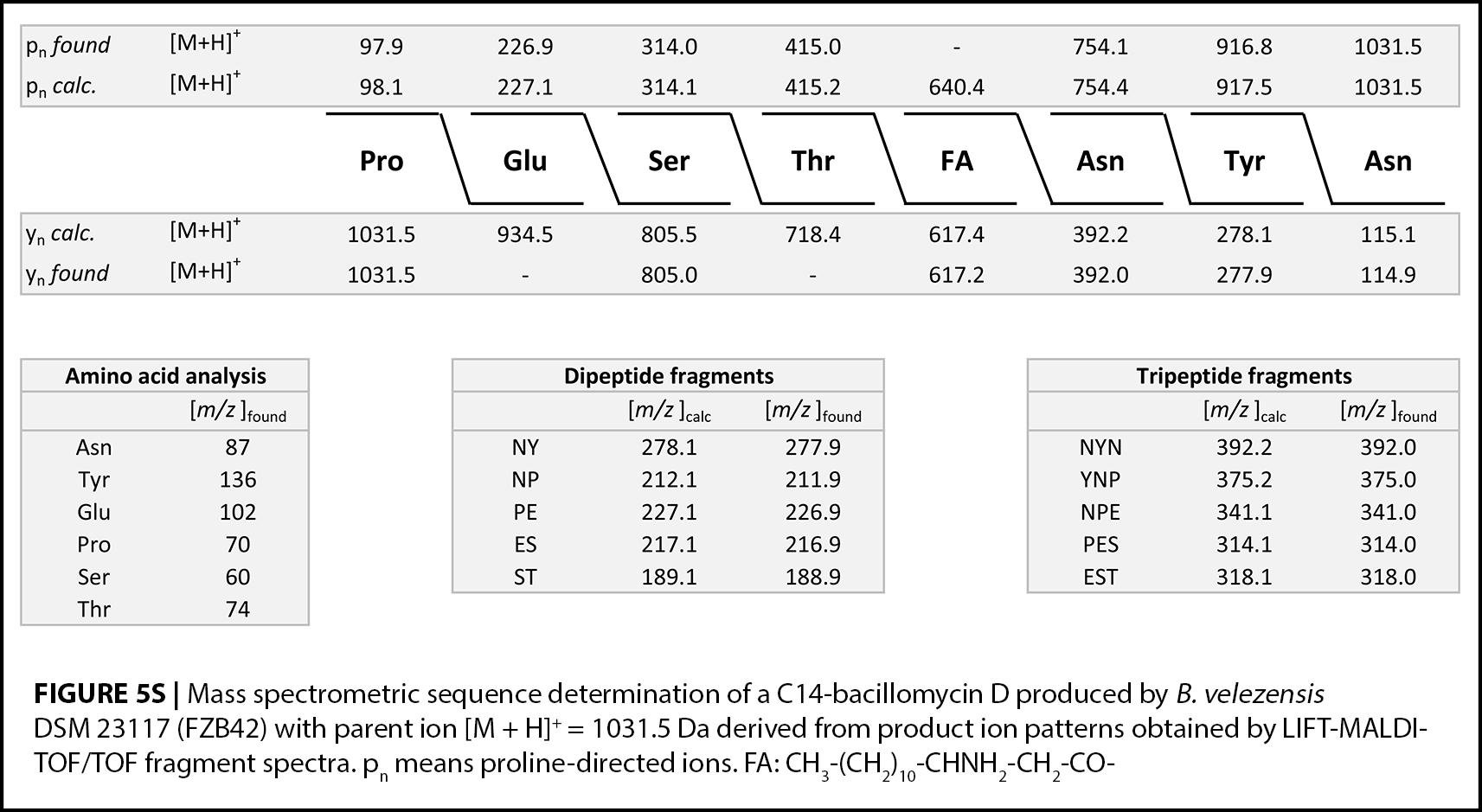

Supplement: Supplementary file 5 [file Image_5.TIF]

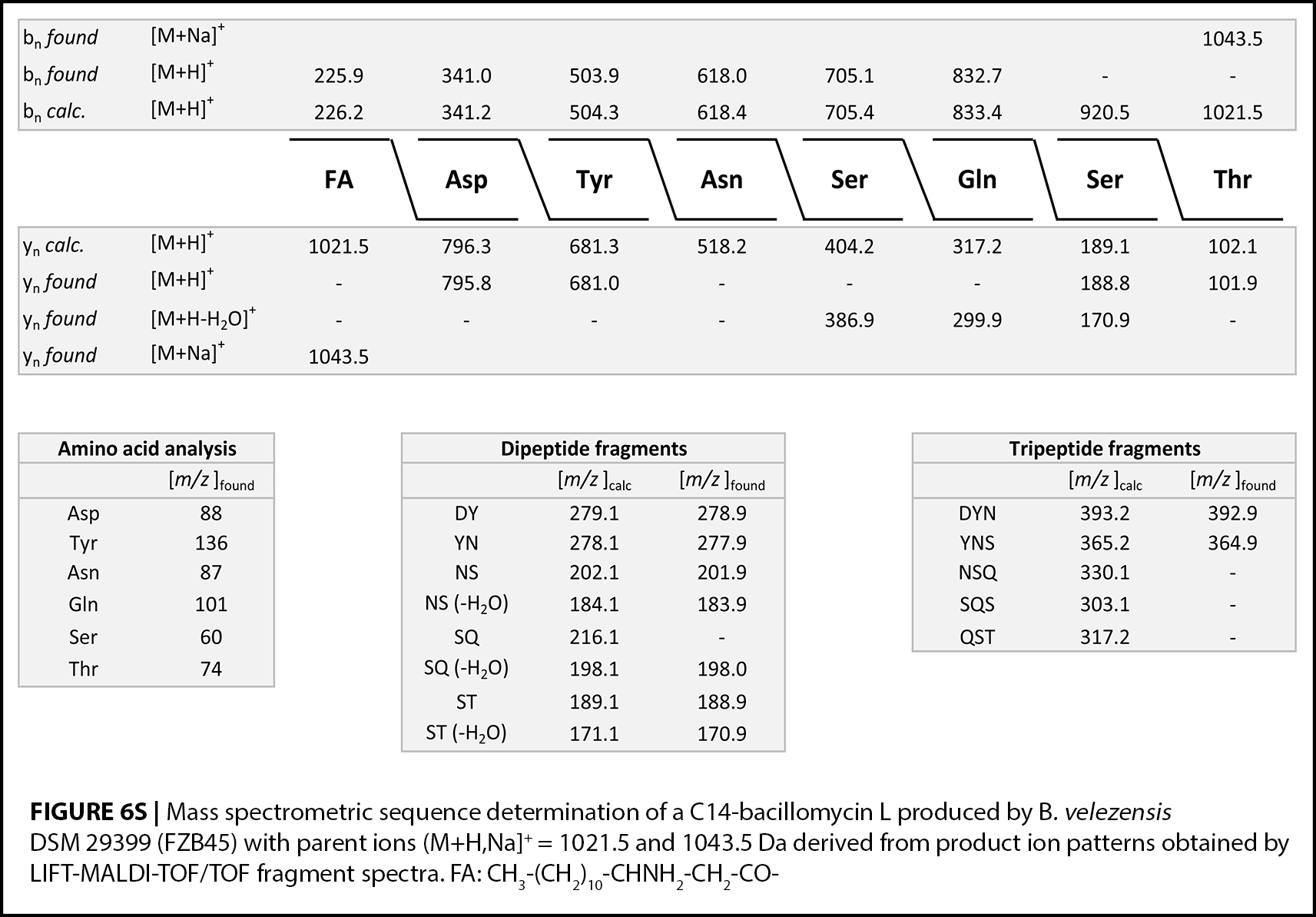

Supplement: Supplementary file 6 [file Image_6.TIF]

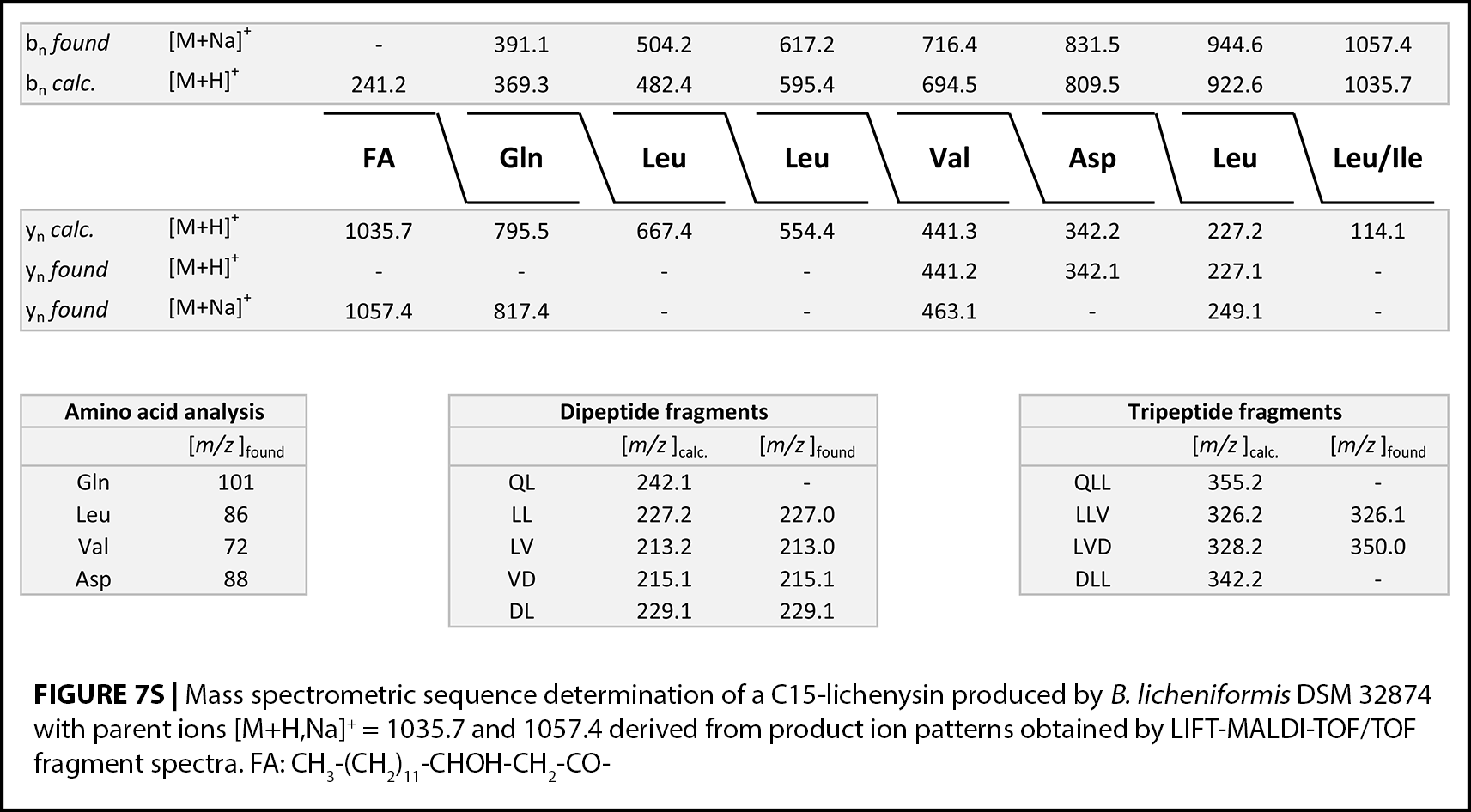

Supplement: Supplementary file 7 [file Image_7.TIF]
